# Comparative efficacy and safety of probiotic for respiratory tract infections in children: a network meta-analysis

**DOI:** 10.3389/fnut.2026.1902533

**Published:** 2026-07-31

**Authors:** Feiyang Na, Yannan Wang, Li Zhou, Baolin Wang, Jiaoyan Lian, Xiaohong Xu

**Affiliations:** 1 Department of Pediatric, Gansu Provincial Maternal and Child-care Hospital (Gansu Provincial Central Hospital), Lanzhou, China; 2 Department of Radiology, Lanzhou University Second Hospital, Lanzhou, China

**Keywords:** probiotic, respiratory tract infections, efficacy, safety, network meta-analysis

## Abstract

**Objective:**

This study aimed to evaluate the comparative efficacy and safety of various probiotic regimens for treating Respiratory tract infections (RTIs) in otherwise healthy children.

**Methods:**

Six Chinese and English databases were searched for randomized controlled trials of probiotics for the RTIs in children published from inception to November 15th, 2025. Pairwise meta-analysis and network meta-analysis were used to assess the efficacy and safety.

**Results:**

Eight RCTs enrolling 1,158 children aged 0.69 to 6.4 years from Ukraine, China, Malaysia, India, and Italy were included. *Bifidobacterium* significantly shortened duration of cough, *Lactobacillus* reduced duration of illness, *Lactobacillus*+*Bifidobacterium* reduced both duration of cough and antibiotic use, and the four-strain combination (*Lactobacillus*+*Bifidobacterium*+*Enterococcus*+*Bacillus*) reduced durations of fever and cough. Low-certainty evidence suggested superior efficacy of the *Lactobacillus*+*Bifidobacterium* over both *Bifidobacterium* and the four-strain combination for reducing duration of cough, and over *Lactobacillus* for shortening antibiotic use. Stratified analyses revealed strain-specific effects across RTI subtypes: *Bifidobacterium* shortened cough in LRTI [−1.12 days, (−1.82, −0.42)]; *Lactobacillus* reduced illness duration in both URTI [−1.31 days, (−2.19, −0.43)] and LRTI [−1.36 days, (−2.29, −0.43)], and shortened antibiotic use in URTI [−1.70 days, (−2.40, −1.00)]; *Lactobacillus*+*Bifidobacterium* shortened fever in URTI [−2.00 days, (−2.57, −1.43)], cough in LRTI [−1.25 days, (−1.84, −0.66)], and both fever −7.10 days, (−8.54, −5.66)] and antibiotic use [−10.50 days, (−13.22, −7.78)] in RRTI; the four-strain combination reduced fever [−1.54 days, (−2.40, −0.68)] and cough [−2.51 days, (−3.89, −1.13)] in RRTI. One RCT reported comparable incidence of gastrointestinal adverse events between probiotic and control groups.

**Conclusion:**

Moderate- to high-certainty evidence supports *Bifidobacterium* for reducing cough duration and *Lactobacillus* for shortening illness duration in children with RTIs. While *Lactobacillus*+*Bifidobacterium* demonstrated large-magnitude reductions in cough duration and antibiotic use, these findings derive from low- to moderate-certainty evidence and require verification through rigorously designed randomized clinical trials. Strain-specific effects varied across RTI subtypes, suggesting the need for tailored probiotic selection. Future research should prioritize validating multi-strain efficacy, establish optimal dosing protocols, and evaluate long-term safety profiles to enable evidence-based guideline development.

**Systematic review registration:**

https://www.crd.york.ac.uk/PROSPERO/view/CRD420251074234, identifier CRD420251074234.

## Introduction

Respiratory tract infections (RTIs), encompassing acute and chronic manifestations, represent a leading cause of global disease burden in pediatric and adult populations, imposing substantial healthcare costs while driving significant morbidity and mortality (1, 2). Lower respiratory tract infections (LRTIs) alone caused 545,700 deaths globally among children under 14 years in 2021, and upper respiratory tract infections (URTIs) contributed to 60% of all-age respiratory mortality (1, 3–6). Although viral etiologies predominate, antibiotic overprescription persists due to perceived parental pressure, clinical time constraints, and practitioner concerns about patient retention-factors that undermine guideline adherence and unnecessarily accelerate antimicrobial resistance (7, 8).

Probiotics, live microorganisms that confer health benefits on the host when administered in adequate amounts, are recommended for preventing or treating several pediatric conditions, including functional gastrointestinal disorders, diarrhea and inflammatory bowel disease (9, 10). Probiotics may modulate respiratory immunity via gut-lung axis pathways, potentially reducing symptom severity and antibiotic dependence. Systematic reviews indicate modest protective effects against RTIs in immunocompetent children, decreasing incidence rates and attenuating symptom severity (11, 12). And, existing randomized controlled trials report conflicting efficacy outcomes, likely due to heterogeneity in probiotic strains, dosing regimens, and patient characteristics. Thus, no comprehensive synthesis has directly compared probiotic interventions across the spectrum of pediatric RTIs.

Systematic reviews employ meta-analyses to synthesize evidence from trials directly comparing two interventions (13). Network meta-analysis (NMA) extends this approach by simultaneously evaluating ≥ 3 competing interventions, leveraging both direct evidence and indirect evidence inferred via shared comparators (13, 14). This NMA therefore evaluates the comparative efficacy and safety of probiotic formulations for pediatric RTIs and determines susceptibility across RTI subtypes based on randomized evidence.

## Methods

This NMA was conducted in accordance with the Preferred Reporting Items for Systematic Reviews and Meta-Analyses (PRISMA) and PRISMA-NMA statements, and registered in the PROSPERO (CRD420251074234) (15, 16).

### Inclusion and exclusion criteria

Study eligibility was defined using the Population, Interventions, Comparators, Outcomes, Study Design (PICOS) framework, as detailed below: (17).

P: previously healthy, ambulatory children aged 6 months to 12 years diagnosed with upper respiratory tract infections (e.g., common cold, pharyngitis) or lower respiratory tract infections (e.g., acute bronchitis, community-acquired pneumonia);

I: orally administered probiotic formulations (any strain, dose, or formulation) initiated after symptom onset;

C: placebo, routine care alone, alternative probiotic regimens, or active controls;

O: clinically oriented endpoints, including duration of core symptoms (i.e., fever, cough), duration of illness, duration of antibiotic use, antibiotic usage rate, and adverse event incidence;

S: randomized controlled trials (RCTs) published in English or Chinese.

Studies were excluded if participants had received systemic antibiotic or probiotic therapy within 1 month prior to enrollment, had a known immunodeficiency disease, had a history of severe allergies (e.g., anaphylaxis), chronic cardiorespiratory, gastrointestinal or other serious systemic disease. Non-human studies, reviews, editorials, editorial material, and errata were also excluded.

### Literature search, screening, and data extraction

A comprehensive and systematic literature search was conducted to identify relevant RCTs investigating the efficacy and safety of probiotics for RTIs in children. Electronic databases including PubMed, Cochrane Central Register of Controlled Trials, Web of Science Core Collection, China National Knowledge Infrastructure, WanFang Data, and Chinese Biomedical Literature Database were searched from inception through November 15th, 2025. The search strategy employed a combination of controlled vocabulary terms and free-text keywords around four conceptual blocks: (1) population: children, pediatric, infant or preschool; (2) intervention: probiotic*, Lactobacillus, Bifidobacterium or Saccharomyces; (3) disease: respiratory tract infection, bronchitis, pneumonia, common cold, or influenza; and (4) study design: randomized controlled trial (Detail information presents in the Supplementary Material Text 1). To minimize publication bias and identify potentially eligible unpublished or ongoing studies, major clinical trial registries (ClinicalTrials.gov, WHO International Clinical Trials Registry Platform, and Chinese Clinical Trial Registry) were screened. Additionally, manual backward citation tracking of included studies and relevant systematic reviews was performed to capture any additional records missed by electronic searches.

All identified records underwent a two-stage screening process conducted independently by two experienced reviewers. Initially, titles and abstracts were assessed against the predefined inclusion and exclusion criteria. Subsequently, full-text articles of potentially eligible studies were retrieved and rigorously evaluated for final inclusion. Any discrepancies between reviewers were resolved through consensus discussion or adjudication by a third senior reviewer.

Following study selection, data extraction was performed independently by the two reviewers using a standardized, pilot-tested electronic form. The extracted data encompassed: (1) study characteristics (author, publication year, country, sample size); (2) participant demographics (age, sex, RTI diagnosis); (3) intervention details (probiotic, dosage, formulation, frequency); (4) comparator information; (5) outcome measures (duration of key symptoms, adverse event rates, antibiotic usage metrics, detailed definitions are provided in Supplementary Material Text 2); and (6) funding sources. Disagreements in data extraction were reconciled by joint re-evaluation of the source documents, with unresolved cases arbitrated by the third reviewer.

### Risk of bias and certainty of evidence assessment

The methodological quality of included RCTs was evaluated independently by two investigators using the Cochrane Risk of Bias tool (RoB) (18). This tool assessed seven domains: (1) random sequence generation, (2) allocation concealment, (3) blinding of participants and personnel, (4) blinding of outcome assessment, (5) incomplete outcome data, (6) selective reporting, and (7) other potential biases. Each domain was judged as “low,” “high,” or “unclear” risk of bias. Discrepancies in ratings were resolved through consensus or consultation with a third reviewer.

The certainty of evidences for each outcome across the NMA was appraised using the Grading of Recommendations Assessment, Development and Evaluate (GRADE) framework for NMAs (GRADE-NMA) (19). This approach evaluated five core domains: (1) RoB, (2) inconsistency, (3) indirectness, (4) imprecision, and (5) publication bias, with additional consideration of network-specific factors (intransitivity and incoherence). Two independent reviewers initially assessed direct evidence estimates for all available comparisons, assigning preliminary certainty ratings based on risk of bias, inconsistency, indirectness, and publication bias. For indirect evidence estimates, the dominant first-order loop connecting interventions was identified, and the preliminary certainty rating was determined by the lowest certainty among direct comparisons constituting this loop. Intransitivity was rated down when serious imbalance existed. The NMA estimate certainty was derived by selecting the higher preliminary certainty rating between direct and indirect estimates as a starting point, followed by assessment of incoherence and imprecision. Final certainty was classified into four levels: high, moderate, low, or very low.

### Data processing and analysis

Extracted outcome data underwent standardized transformations to ensure consistency for quantitative synthesis. Standard errors (SE) were converted to standard deviations (SD) using the formula SD = SE × $\sqrt{n}$, where n is the sample size. For studies reporting medians and interquartile ranges (IQR), mean and SD values were estimated using validated methods by Cochrane Handbook (20).

Pairwise meta-analysis or NMA were conducted within a frequentist framework using the netmeta package (version 2.7-0) in R software (version 4.3.1). Continuous outcomes were analyzed as mean differences (MD) with 95% confidence intervals (CI), while dichotomous outcomes were analyzed as odds ratio (OR) with 95% CI. To address potential bias from small-study effects and heterogeneity, the DerSimonian-Laird method with the Hartung-Knapp-Sidik-Jonkman (HKSJ) method was applied to adjust CI widths for all summary estimates (21, 22). Global heterogeneity was quantified using the I^2^ statistic and Cochran’s Q test, with I^2^ > 50% indicating substantial heterogeneity. Consistency between direct and indirect evidence was evaluated via node-splitting and design-by-treatment interaction models. Publication bias was assessed using Comparison-adjusted funnel plot when at least ten studies were included. To evaluate the robustness of primary findings, prespecified subgroup analyses were conducted by stratifying the network according to respiratory infection categories: URTIs, LRTIs, and recurrent respiratory tract infections (RRTIs). In addition, we conducted sensitivity analyses by using DL model without HKSJ, fixed effects models, and excluding studies with attrition rates exceeding 20%. The “networkplot” function of STATA SE software (Version 15) was utilized to draw network plots describing and presenting visually the strength of the associations on different intervention.

## Results

**FIGURE 1:**
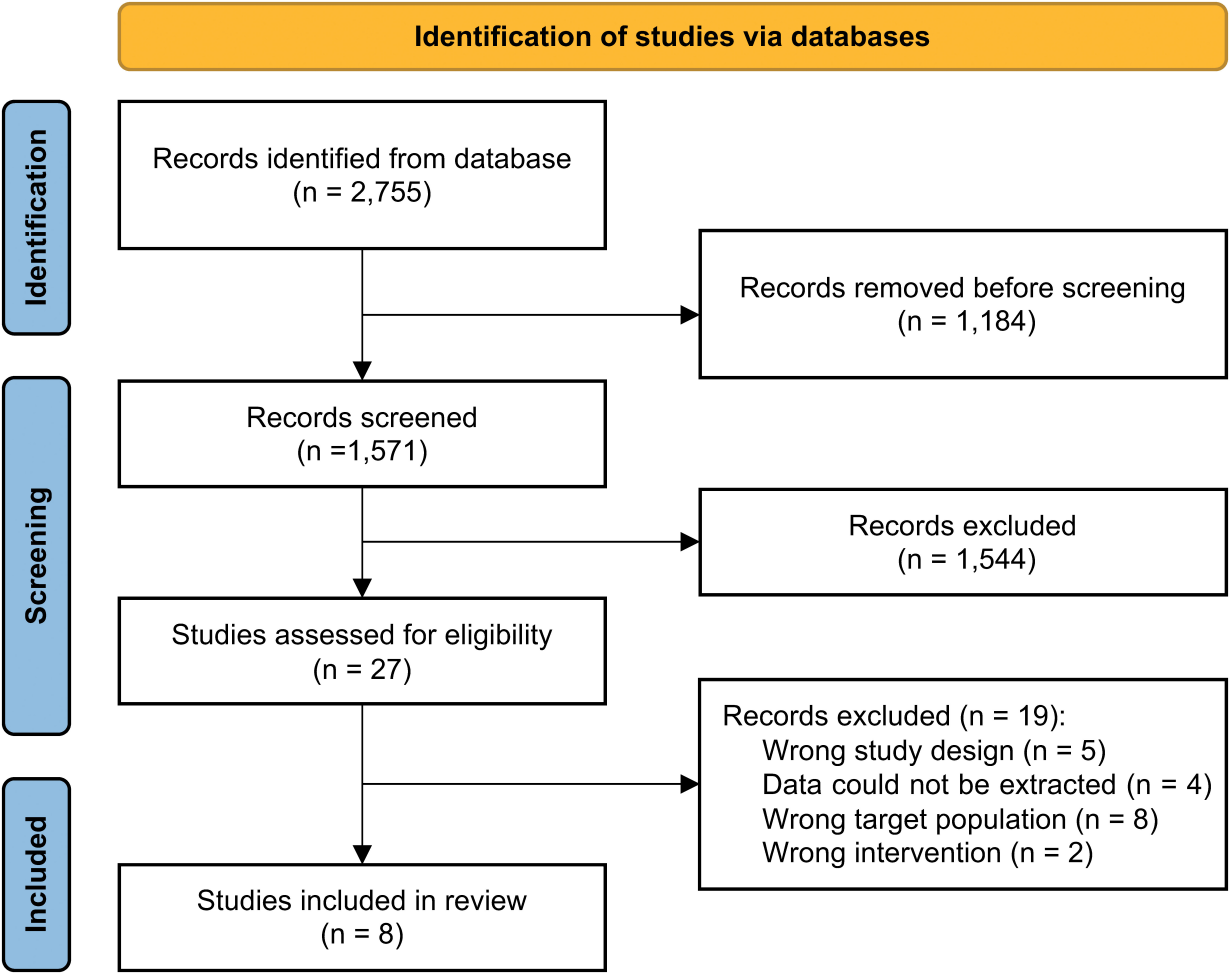
Study selection.

2,769 records were retrieved. After removing duplicates, 1,541 articles underwent screening based on titles and abstracts. Full texts of 27 potentially eligible studies were reviewed, and ultimately 8 studies were included (23–30) (Figure 1). The list of excluded studies is presented in Supplementary Material Text 3.

### Basic Characteristics, risk of bias, and certainty of evidence results

**FIGURE 2:**
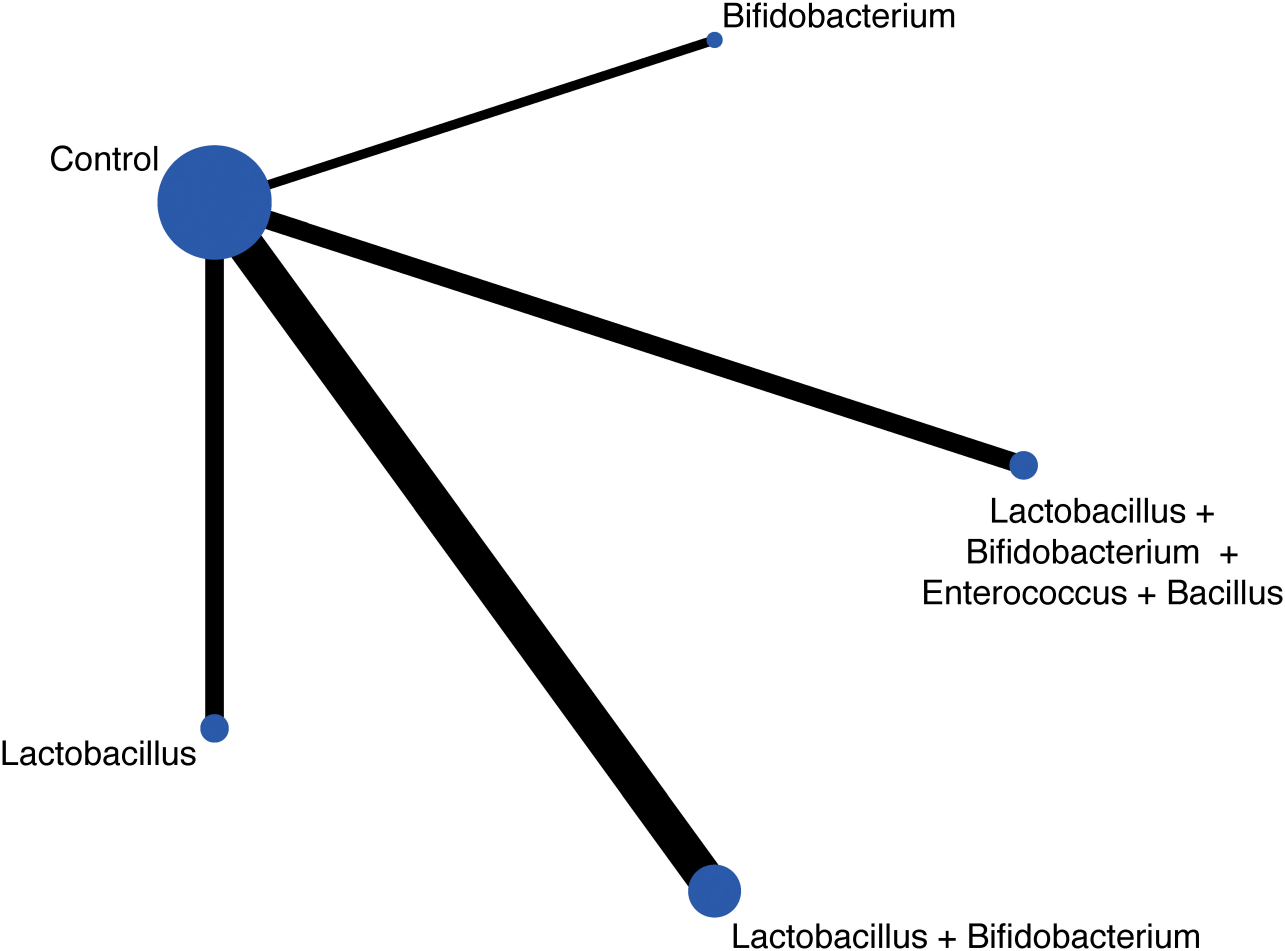
Network plots of all included studies.

This study incorporated eight RCTs (23–30), encompassing a total of 1,158 pediatric participants. The studies were conducted across diverse geographical regions including China (*n* = 4), Ukraine (*n* = 1), Malaysia (*n* = 1), India (*n* = 1), and Italy (*n* = 1). Participants ranged from infants (0.69 ± 0.10 years old) to school-aged children (6.50 ± 2.79 years old), with female representation ranging from 35 to 53%. Interventions primarily consisted of various multi-strain probiotic formulations (Lactobacillus, Bifidobacterium, often combined with Enterococcus and/or Bacillus), administered in powder or tablet form at doses ranging from 3 million to 20 billion colony-forming units (CFU) daily, over durations of 1 to 3 times per day. Detailed information is presented in Table 1. Figure 2 presents the sample size (node size) and the number of included studies (line thickness).

**TABLE 1:** Characteristics of included studies.

Study ID	Country	Funding sources	Sample size (Intervention/Control)	Age (Years)	Female (%)	Type of RTI	Type of disease	Interventions/ Controls	Main results	Drop-out rate
Gerasimov, (23)	Ukraine	Health Ministry of Ukraine	137 (64/73)	6.50 ± 2.79	47%	Acute respiratory infection	Unspecified^a^ Non-recurrent	Lactobacillus + Bifidobacterium, 5 billion CFU, powder, 1 time/day Placebo	Illness duration was significantly shortened (median 5.0 vs. 7.0 days, *p* < 0.001), and disease severity was lower (240 vs. 525 score-days, *p* < 0.001). Antibiotic prescription rates were comparable (13% vs. 11%); duration of use was not reported separately.	43%
Huo, (24)	China	Department of Health of Hainan Province	85 (43/42)	4.55 ± 1.99	45%	Recurrent respiratory infections	Unspecified^a^ Recurrent	Lactobacillus + Bifidobacterium, 3 million CFU, tablet, 3 times/day Placebo	Core symptoms were significantly reduced: fever (6.4 ± 2.3 vs. 13.5 ± 4.2 days) and cough (11.6 ± 4.6 vs. 20.3 ± 6.7 days), both *p* < 0.05. Total illness duration was significantly shorter (13.2 ± 6.0 vs. 23.4 ± 7.8 days). Antibiotic duration of use was significantly reduced (12.1 ± 5.6 vs. 22.6 ± 7.1 days). Only one case of mild constipation was reported in the probiotic group, which did not affect treatment continuation.	0%
Li, (25)	China	Qingdao Outstanding Health Professional Development Fund	60 (30/30)	3.81 ± 2.26	53%	Recurrent respiratory infections	Unspecified^a^ Recurrent	Lactobacillus + Bifidobacterium + Enterococcus + Bacillus, tablet, 2 times/day Routine Treatment	Core symptoms per episode were significantly reduced: fever (2.03 ± 1.45 vs. 3.57 ± 1.93 days) and cough (4.02 ± 1.85 vs. 6.53 ± 3.39 days), both *p* < 0.01. Antibiotic use was significantly reduced in both frequency (0.63 ± 1.47 vs. 2.17 ± 3.57 episodes/year, *p* = 0.0159) and duration per episode (4.13 ± 2.54 vs. 6.12 ± 2.87 days, *p* = 0.0031).	0%
Mageswary, (26)	Malaysia	Universiti Sains Malaysia	120 (60/60)	1.08 ± 1.08	35%	Acute respiratory tract infections	Both^b^ Non-recurrent	Bifidobacterium, 20 billion CFU, powder, 1 time/day Placebo	Core symptoms were significantly reduced in duration during hospitalization and at 4 weeks post-discharge (all *p* < 0.05), with more pronounced benefits in LRTI patients. Hospitalization > 7 days was significantly prevented in antibiotic-prescribed patients (0% vs. 7.69%, *p* = 0.004). Antibiotic prescriptions at discharge were significantly reduced (15.0% vs. 26.7%, *p* = 0.037), and new prescriptions in non-prescribed patients were prevented (*p* = 0.024).	12%
Zhang, (27)	China	NR	114 (57/57)	0.69 ± 0.10	42%	Bronchiolitis	Lower respiratory tract Non-recurrent	Lactobacillus + Bifidobacterium + Enterococcus + Bacillus, tablet, 3 times/day Routine Treatment	Cough resolved significantly earlier (7.25 ± 1.52 vs. 8.50 ± 1.68 days, *p* = 0.021); disappearance times for wheezing, pulmonary rales, and retractions were also significantly shorter than in the control group (all *p* < 0.05). Illness duration was consistently reduced.	0%
Pulmamidi, (28)	Telangana	NR	388 (200/200)^c^	4.93 ± 0.56	50%	Recurrent respiratory infections	Both^b^ Recurrent	Lactobacillus + Bifidobacterium, 1 time/day Placebo	Cough duration was significantly reduced (11.9 ± 3 vs. 23.5 ± 5 days, *p* = 0.006). Total URTI symptom duration was significantly shortened (22 ± 4 vs. 43 ± 32 days, *p* = 0.006).	3%
He, (29)	China	NR	84 (44/40)	5.62 ± 1.85	50%	Acute upper respiratory tract infections	Upper respiratory tract Non-recurrent	Lactobacillus, 1 billion CFU, tablet, 2 times/day Routine Treatment	Cure time within 7 days was significantly shorter for acute URTI (3.83 vs. 5.14 days) and acute bronchitis (4.28 vs. 5.64 days), both p < 0.01; the 21-day illness course was also significantly shortened (both *p* < 0.05). Antibiotic use rate at 7 days was significantly reduced in acute bronchitis (74.4% vs. 95.0%, *p* = 0.010); duration of antibiotic use at 7 days was significantly shortened in acute URTI (3.22 vs 4.92 days, *p* < 0.001). Safety: only 2/43 (4.7%) probiotic recipients reported mild cough due to improper timing with antibiotics.	20%
			83 (43/40)	5.28 ± 1.86	42%	Acute bronchitis	Lower respiratory tract Non-recurrent			16%
Bettocchi, (30)	Italy	Italian Ministry of Health, Coree Srl.	87 (37/50)	2.55 ± 1.30	46%	Upper respiratory tract infections	Upper respiratory tract Non-recurrent	Lactobacillus + Bifidobacterium, 3 billion CFU, powder or oily, 1 time/day Placebo	Fever duration was significantly shortened: median 3 days (IQR 2-4) vs 5 days (IQR 4-6). Antibiotic prescriptions after discharge did not differ significantly (2% vs. 12%, *p* = 0.16). Safety: adverse events were comparable between groups, including constipation (16% vs. 12%) and abdominal pain (8% vs. 4%), with all *p* > 0.05.	32%

*CFU, colony-forming unit; NR, not report.

^a^Study does not specify between upper or lower respiratory tract infections.

^b^The study included both upper and lower respiratory tract infections.

^c^Three children withdrew from the research and failed to provide any documentation, nine children were left out of the per-protocol analysis (three because the follow-up period was not completed; six because therapy was used without authorization).

Most studies demonstrated low risk concerning random sequence generation (*n* = 6) and selective outcome reporting (*n* = 8). However, allocation concealment was frequently unclear (*n* = 5). Blinding of participants/personnel and outcome assessors presented limitations, with half of the studies rated as unclear for one or both domains. Incomplete outcome data was generally low risk (*n* = 6), although two trials (23, 29) exhibited high risk due to substantial dropout rates (43% and 32%, respectively). Assessment for other potential sources of bias was predominantly low or unclear (both *n* = 4). Detailed information is presented in Supplementary Table 1.

Among 21 comparisons for the five outcomes, one was rated as high certainty, three as moderate certainty, eight as low certainty, and nine as very low certainty. The results of certainty of evidence are presented in Supplementary Material Text 4–8.

### Efficacy

**FIGURE 3:**
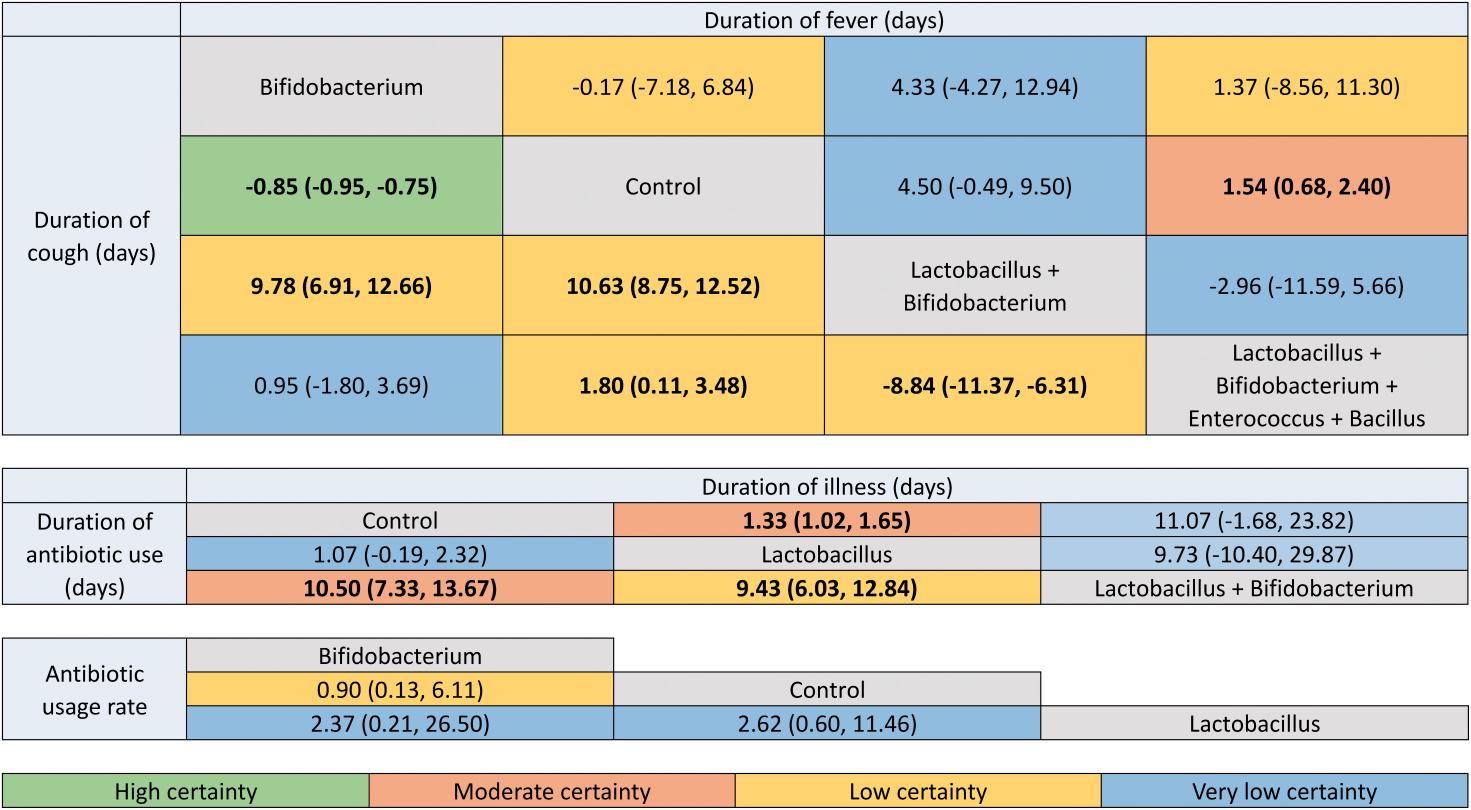
League table of all outcome related of efficacy.

**FIGURE 4:**
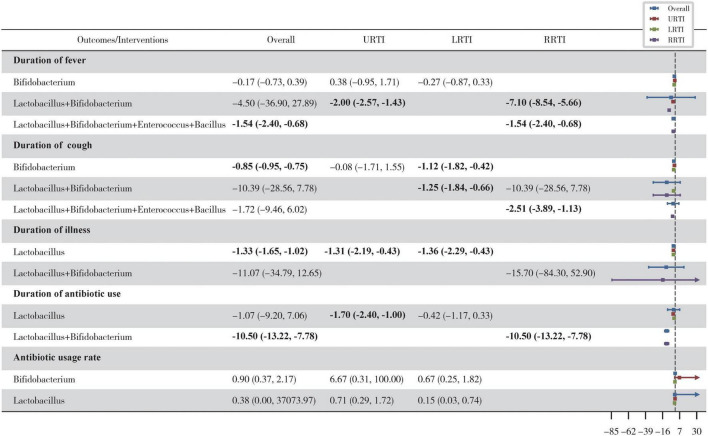
The results of stratified analysis of different types of respiratory infections.

Compared with the control group, Bifidobacterium shortened the duration of cough [MD = −0.85 days, 95% CI (−0.95, −0.75), high certainty], Lactobacillus shortened the duration of disease [−1.33 days, (−1.65, −1.02), moderate certainty], Lactobacillus + Bifidobacterium shortens duration of cough [−10.63 days, (−12.52, −8.75), low certainty] and duration of antibiotic use [−10.50 days, (−13.67, −7.33), moderate certainty], four-strain combination (Lactobacillus + Bifidobacterium + Enterococcus + Bacillus) shortens duration of fever [−1.54 days, (−2.40, −0.68), moderate certainty] and duration of cough [−1.80 days, (−3.48, −0.11), low certainty]. And low certainty evidence suggests that Lactobacillus + Bifidobacterium is also significantly superior to Bifidobacterium and four-strain combination in shortening the duration of cough, and better than Lactobacillus in shortening the duration of antibiotic use (Figure 3). Stratified analysis showed that Bifidobacterium shortened the duration of cough of LRTI [−1.12 days, (−1.82, −0.42)], Lactobacillus shortened the duration of illness of URTI pediatrics [−1.31 days, (−2.19, −0.43)] and duration of antibiotic use [−1.70 days, (−2.40, −1.00)], and the duration of illness of LRTI pediatrics [−1.36 days, (−2.29, −0.43)]. Lactobacillus + Bifidobacterium shortened the duration of fever of URTI pediatrics [−2.00 days, (−2.57, −1.43)], the duration of cough of LRTI [−1.25 days, (−1.84, −0.66)], and the duration of fever [−7.10 days, (−8.54, −5.66)] and antibiotic use [−10.50 days, (−13.22, −7.78)] of RRTI. Four-strain combination shortened the duration of fever in children with RRTI [−1.54 days, (−2.40, −0.68)] and the duration of cough [−2.51 days, (−3.89, −1.13)] (Figure 4). The forest plot is presented in Supplementary Figures 1–5. Network plots for each outcomes are presented in Supplementary Figures 6–10.

### Safety

One study (30) reported the presence of three gastrointestinal adverse events, including diarrhea (4 cases in the control group), constipation (6 cases in both *Lactobacillus* + *Bifidobacterium* and control group) and abdominal pain (3 cases in *Lactobacillus* + *Bifidobacterium* group and 2 cases in the control group).

### Additional analysis

No publication bias was detected because the number of included studies was less than 10 for all outcomes. The results of sensitivity analysis by using different models are shown in Supplementary Tables 2–6, and by excluding studies with attrition rates exceeding 20% are shown in Supplementary Tables 7–9. None of the outcomes reported statistically significant differences in global inconsistency due to the small number of included studies.

## Discussion

This NMAs comprehensively evaluated the efficacy and safety of probiotic monotherapies and combinations in children with RTIs. Eight eligible RCTs involved 1,158 participants assessed outcomes including duration of cough, fever, illness, antibiotic use, antibiotic usage rate, and adverse events. *Bifidobacterium* significantly shortened cough duration with high-certainty evidence, which was consistent across sensitivity analyses and stratified by RTI subtype, where *Bifidobacterium* demonstrated particular efficacy in LRTI. The magnitude of effect, while modest, is clinically relevant given the high prevalence of cough as a burdensome symptom in pediatric RTIs and the absence of specific antiviral therapies targeting cough resolution. *Lactobacillus* reduced overall duration of illness with moderate-certainty evidence, which revealed consistent effects across URTI and LRTI, suggesting broad-spectrum efficacy across RTI types. Notably, the 1.70-day reduction per URTI episode represents a clinically meaningful decrease in antibiotic exposure, particularly in children with recurrent infections, as it reduces the cumulative risk of antibiotic-associated adverse effects and selective pressure for antimicrobial resistance. This finding aligns with the emerging paradigm that probiotics may mitigate the need for antibiotics by accelerating clinical recovery and reducing symptom severity.

The *Lactobacillus* + *Bifidobacterium* demonstrated the largest effect magnitudes across all outcomes, with reductions in cough duration and antibiotic use duration. However, these estimates are derived from low- to moderate-certainty evidence and warrant careful interpretation. The exceptionally large effect for cough duration is primarily driven by Pulmamidi et al. (28) in which cough was measured as cumulative cough days over a 6-month intervention period, rather than as per-episode duration. This methodological distinction, cumulated burden versus single-episode duration, explains the apparent biological implausibility of a 10.6-day reduction in a disease with typical per-episode duration of 7–14 days. Stratified analyses further elucidated the subtype-specific efficacy of this combination: it shortened fever in URTI, cough in LRTI, and both fever and antibiotic use in RRTI. The most pronounced effects observed in RRTI populations suggest that probiotics confer the greatest absolute benefit in children with the highest cumulative antibiotic burden, supporting their role as a targeted antimicrobial stewardship intervention in this high-risk subgroup.

The four-strain combination reduced fever duration with moderate-certainty evidence and cough duration with low-certainty evidence. Stratified by RTI subtype, this combination reduced fever and cough specifically in RRTI, suggesting that broader strain diversity may confer additional benefit in children with recurrent infections, potentially through enhanced gut-lung axis modulation and immune training.

Safety data from one RCT reported comparable incidence of gastrointestinal adverse events (diarrhea, constipation, abdominal pain) between probiotic and control groups. These findings, while reassuring, are derived from a single study and do not adequately characterize the safety profile of other probiotic formulations. Given the theoretical risk of antibiotic resistance gene transfer from probiotic strains to pathogenic gut commensals, a concern raised in recent systematic reviews, future trials should incorporate metagenomic surveillance of the gut resistome as a standard safety endpoint, particularly in vulnerable pediatric populations.

Our findings extend and complement several recently published systematic reviews and meta-analyses on biotics for respiratory and related infections. Zhao et al demonstrated that probiotics reduce the risk of experiencing at least one acute URTI episode and shorten the mean duration, with a moderate-certainty reduction in antibiotic prescriptions (31). Whereas their review focused on prevention in community-based populations, our NMA addresses the t use of probiotics in children with established RTIs. Importantly, our strain-level hierarchy, showing *Bifidobacterium* as most effective for cough duration and *Lactobacillus* for overall illness duration, provides novel granularity not available in the Cochrane synthesis, which pooled all strains together. Thus, our NMA offers a complementary evidence base for clinicians deciding which specific probiotic to prescribe once an infection has occurred, whereas the Cochrane review supports population-level preventive strategies. Coleman et al reported that probiotics reduced RTI incidence, total illness days, duration and severity, with effect modifiers including physical activity and fermented-dairy delivery (32). The overall direction and magnitude of effect are broadly consistent with our pediatric NMA. However, their subgroup analyses did not detect statistically significant differences between *Lactobacillus*- and *Bifidobacterium*-based interventions, whereas our NMA clearly distinguished strain-specific efficacy. This discrepancy may reflect age-related differences in gut microbiota composition, immune maturation, or disease aetiologia, underscoring the importance of population-specific evidence synthesis. Furthermore, their identification of fermented dairy as a beneficial delivery matrix was not systematically examined in our included pediatric trials, representing a promising avenue for future formulation research. Fan et al reviewed microbiota-targeted therapies in pediatric asthma, finding that *Lactobacillus* and *Bifidobacterium* reduce asthma exacerbations and improve pulmonary function, while bacterial lysates attenuate airway hyperresponsiveness (33). Although our NMA focused on acute RTIs rather than chronic asthma, the convergent findings across acute and chronic respiratory conditions are noteworthy. Mechanistically, probiotics may modulate the gut-lung axis through shared pathways, Th1/Th2 balance restoration, regulatory T-cell induction, and short-chain fatty acid production, that provide both immediate symptom relief during acute infections and long-term immune tolerance in atopic children. This suggests that microbiota-targeted interventions could have dual utility: shortening acute episodes while potentially modifying the trajectory toward recurrent wheeze or asthma, though this hypothesis requires dedicated longitudinal trials. Regarding antimicrobial resistance, Mazandarani et al concluded that adjunctive probiotics and symbiotic improve eradication rates, reduce infection recurrence, and decrease antibiotic use, with most studies reporting positive effects on resistance phenotypes (34). Our NMA directly supports this conclusion, as we observed significantly shorter antibiotic use duration and lower antibiotic usage rates in several probiotic groups, particularly the *Lactobacillus* + *Bifidobacterium* combination. By reducing the total antibiotic exposure per episode, probiotics may alleviate the selective pressure that drives resistance emergence, a benefit that aligns with antimicrobial stewardship goals.

In our included studies, probiotics were generally well-tolerated, with gastrointestinal adverse events occurring at rates comparable to placebo, consistent with prior safety syntheses. However, as highlighted by Mazandarani et al. (34) and other recent commentaries, the use of live bacterial preparations is not entirely without risk. A growing body of evidence indicates that probiotic strains, particularly *Lactobacillus* and *Bifidobacterium*, can harbor intrinsic or acquired antibiotic resistance genes. These genes may be transferred horizontally to pathogenic enterobacteria or to the resident gut microbiota via conjugation, transduction, or transformation, potentially compounding the very resistance problem that probiotics are intended to mitigate (34–36). Several factors moderate this concern in the context of pediatric RTIs. First, the interventions in our included studies were short-term (typically 7–14 days, with some up to 3 months), which limits the duration of exposure and the opportunity for sustained colonization and gene transfer. Second, the clinical benefits, shortened illness, reduced antibiotic courses, may outweigh theoretical risks in otherwise healthy children, particularly when probiotics are used as adjuncts to standard care rather than as long-term prophylaxis. Third, none of the included trials reported systemic infections attributable to probiotics, which aligns with the low baseline risk in immunocompetent pediatric populations. Nevertheless, we acknowledge that routine safety monitoring in current RCTs rarely includes metagenomic surveillance of the gut resistome. Future trials should incorporate pre- and post-intervention stool metagenomics to track ARG abundance and diversity, particularly in vulnerable subgroups (e.g., immunocompromised children, those with central venous catheters, or those receiving prolonged broad-spectrum antibiotics). Until such data emerge, we concur with Mazandarani et al. (34) that clinicians should exercise caution in high-risk settings and consider strain-specific resistance profiles when selecting a probiotic product. Regulatory bodies may also benefit from standardized minimum safety requirements, including ARG screening, for all probiotic strains intended for pediatric use. This balanced approach, acknowledging both the robust efficacy signals and the emerging safety caveats, will guide responsible clinical translation and inform future research priorities.

This NMA provides evidence to inform probiotic selection for pediatric RTIs while highlighting critical knowledge gaps. Clinically, *Bifidobacterium* may be prioritized for duration of cough reduction given high-certainty evidence, while *Lactobacillus* shows moderate-certainty evidence for shortening duration of illness, both offering feasible single-strain options where formulary restrictions exist. The pronounced effects of *Lactobacillus* + *Bifidobacterium* on duration of cough and antibiotic use, though derived from low-certainty evidence, warrant consideration in antimicrobial stewardship programs where reducing antibiotic exposure is paramount; however, clinical implementation requires caution pending verification through adequately powered RCTs. Future research must prioritize: 1) dose-optimization trials for multi-strain formulations to reconcile efficacy-safety balances, 2) pragmatic studies evaluating real-world adherence and cost-effectiveness, particularly for resource-limited settings, and 3) standardized safety monitoring across probiotic strains to address current evidence sparsity. Guideline committees should conditionally recommend high/moderate-certainty interventions while designating multi-strain combinations as “only in research” until robust efficacy replication and long-term safety data emerge, a staged approach that aligns evidence maturity with clinical urgency while mitigating prescription uncertainties.

To our knowledge, present study is the first NMA to comprehensive evaluation the efficacy and safety of different probiotic strains in children with RTIs. We conducted a systematic literature search, rigorous literature screening, data extraction, and quality assessment, and used the GRADE method to assess the certainty of evidence, providing reliability for the study results. Nevertheless, our study has some limitations. Firstly, high/unclear bias in allocation concealment (5/8 trials) and variable dropout (0–43%) may inflate effect sizes. However, node-splitting showed no inconsistence. Secondly, dosing heterogeneity (3 million–20 billion CFU) precludes optimal regimen standardization, and due to the small number of studies included, it is not possible to further explore the effects of different dosages or the efficacy at the strain level in compound preparations using methods such as meta-regression. Thirdly, although some outcomes were rated as having moderate or high certainty, the number of included studies was small, and large numbers of well-designed, high-quality clinical studies are still needed in the future to validate the findings.

## Conclusion

This study demonstrates that probiotic interventions confer strain-specific benefits in pediatric respiratory tract infections. Moderate- to high-certainty evidence supports *Bifidobacterium* for reducing cough duration and *Lactobacillus* for shortening overall illness duration and antibiotic use in URTI. The *Lactobacillus* + *Bifidobacterium* shows the largest effect magnitudes for cough and antibiotic use reduction, particularly in recurrent infections, though these findings derive from low- to moderate-certainty evidence and require validation. Safety data indicate a favorable short-term profile. Current evidence supports the selective use of high/moderate-certainty probiotic regimens in clinical practice to mitigate symptom burden, with potential applications in antimicrobial stewardship programs. Future research should prioritize validating multi-strain efficacy, establish optimal dosing protocols, and evaluate long-term safety profiles to enable evidence-based guideline development and resolve existing prescription uncertainties.

## Data Availability

The original contributions presented in this study are included in this article/Supplementary material, further inquiries can be directed to the corresponding author.
