# Supplementary material for: Comparative efficacy and safety of probiotic for respiratory tract infections in children: a network meta-analysis
Source: Front Nutr. 2026 Jul 31;13:1902533. doi: 10.3389/fnut.2026.1902533 (PMC13473285; doi:10.3389/fnut.2026.1902533)
Supplement: Supplementary file 1 [file Table_1.docx]

**Text S1 Search strategy**

PubMed

#1 "Respiratory Tract Infections"[Mesh] OR "Common Cold"[Mesh] OR "Sinusitis"[Mesh] OR "Pharyngitis"[Mesh] OR "Laryngitis"[Mesh] OR "Parainfluenza Virus 2, Human"[Mesh] OR "Tonsillitis"[Mesh] OR "Peritonsillar Abscess"[Mesh] OR "Croup"[Mesh] OR "Epiglottitis"[Mesh] OR "Supraglottitis"[Mesh] OR "Otitis Media"[Mesh] OR "Pneumonia"[Mesh] OR "Bronchopneumonia"[Mesh] OR "Bronchitis"[Mesh] OR "Pleurisy"[Mesh] OR "Rhinitis"[Mesh] OR "Rhinosinusitis"[Mesh] OR Respiratory Infection[Title/Abstract] OR Upper Respiratory Infection[Title/Abstract] OR Lower Respiratory Infection[Title/Abstract] OR Respiratory Tract Infection[Title/Abstract] OR Common Cold[Title/Abstract] OR Sinusitis[Title/Abstract] OR Pharyngitis[Title/Abstract] OR Laryngitis[Title/Abstract] OR Laryngotracheobronchitis[Title/Abstract] OR Tonsillitis[Title/Abstract] OR peritonsillar abscess[Title/Abstract] OR Croup[Title/Abstract] OR Epiglottitis[Title/Abstract] OR supraglottitis[Title/Abstract] OR Pneumonia[Title/Abstract] OR Bronchopneumonia[Title/Abstract] OR Bronchitis[Title/Abstract] OR Pleurisy[Title/Abstract] OR Pleuritis[Title/Abstract] OR rhinitis[Title/Abstract] OR rhinosinusitis[Title/Abstract]

#2 "Probiotics"[Mesh] OR "Lactobacillus"[Mesh] OR "Bifidobacterium"[Mesh] OR "Lactococcus"[Mesh] OR "Saccharomyces"[Mesh] OR "Streptococcus thermophilus"[Mesh] OR "Bacillus subtilis"[Mesh] OR "Enterococcus"[Mesh] OR probiotic*[Title/Abstract] OR lactobacill*[Title/Abstract] OR bifido*[Title/Abstract] OR bifidu*[Title/Abstract] OR lactococc*[Title/Abstract] OR saccharomyc*[Title/Abstract] OR streptococcus thermophilus[Title/Abstract] OR bacillus subtilis[Title/Abstract] OR enterococcus faec*[Title/Abstract] OR bulgarian bacillus[Title/Abstract]

#3 "Infant"[Mesh] OR "Child"[Mesh] OR "Pediatrics"[Mesh] OR child*[Title/Abstract] OR early life[Title/Abstract] OR paediatric*[Title/Abstract] OR pediatric*[Title/Abstract] OR preschool*[Title/Abstract] OR pre-school*[Title/Abstract] OR infant*[Title/Abstract]

#4 "clinical trials, phase ii as topic"[MeSH Terms] OR "clinical trials, phase iii as topic"[MeSH Terms] OR "clinical trials, phase iv as topic"[MeSH Terms] OR "clinical trials as topic"[MeSH Terms] OR "Controlled Clinical Trials as Topic"[MeSH Terms] OR "Randomized Controlled Trials as Topic"[MeSH Terms] OR "Intention to Treat Analysis"[MeSH Terms] OR "Pragmatic Clinical Trials as Topic"[MeSH Terms] OR "controlled clinical trial"[Publication Type] OR "randomized controlled trial"[Publication Type] OR "clinical trial"[Publication Type] OR "Single-Blind Method"[MeSH Terms] OR "Double-Blind Method"[MeSH Terms] OR "RCT"[Title/Abstract] OR "random*"[Title/Abstract] OR "placebo"[Title/Abstract] OR "controlled"[Title/Abstract] OR "blind*"[Title/Abstract] OR "single blind*"[Title/Abstract] OR "double blind*"[Title/Abstract] OR "triple blind*"[Title/Abstract]

#5 #1 AND #2 AND #3 AND #4

#6 #5 AND Filters: Chinese, English, Humans

Cochrane Library

#1 MeSH descriptor: [Respiratory Tract Infections] explode all trees

#2 MeSH descriptor: [Common Cold] explode all trees

#3 MeSH descriptor: [Pharyngitis] explode all trees

#4 MeSH descriptor: [Parainfluenza Virus 2, Human] explode all trees

#5 MeSH descriptor: [Tonsillitis] in all MeSH products

#6 MeSH descriptor: [Peritonsillar Abscess] explode all trees

#7 MeSH descriptor: [Croup] explode all trees

#8 MeSH descriptor: [Epiglottitis] explode all trees

#9 MeSH descriptor: [Supraglottitis] explode all trees

#10 MeSH descriptor: [Pneumonia] explode all trees

#11MeSH descriptor: [Bronchopneumonia] explode all trees

#12 MeSH descriptor: [Bronchitis] explode all trees

#13 MeSH descriptor: [Pleurisy] explode all trees

#14 MeSH descriptor: [Rhinitis] explode all trees

#15 MeSH descriptor: [Rhinosinusitis] explode all trees

#16 MeSH descriptor: [Sinusitis] explode all trees

#17 MeSH descriptor: [Laryngitis] explode all trees

#18 (Respiratory Infection OR Upper Respiratory Infection OR Lower Respiratory Infection OR Respiratory Tract Infection OR Common Cold OR Sinusitis OR Pharyngitis OR Laryngitis OR Laryngotracheobronchitis OR Tonsillitis OR peritonsillar abscess OR Croup OR Epiglottitis OR supraglottitis OR Otitis Media OR Pneumonia OR Bronchopneumonia OR Bronchitis OR Pleurisy OR Pleuritis OR rhinitis OR rhinosinusitis):ti,ab,kw

#19 #1 OR #2 OR #3 OR #4 OR #5 OR #6 OR #7 OR #8 OR #9 OR #10 OR #11 OR #12 OR #13 OR #14 OR #15 OR #16 OR #17 OR #18

#20 MeSH descriptor: [Probiotics] explode all trees

#21 MeSH descriptor: [Lactobacillus] explode all trees

#22 MeSH descriptor: [Bifidobacterium] explode all trees

#23 MeSH descriptor: [Saccharomyces] explode all trees

#24 MeSH descriptor: [Streptococcus thermophilus] explode all trees

#25 MeSH descriptor: [Bacillus subtilis] explode all tree

#26 MeSH descriptor: [Enterococcus] explode all trees

#27 MeSH descriptor: [Lactococcus] explode all trees

#28 (probiotic* OR lactobacill* OR bifido* OR bifidu* OR lactococc* OR saccharomyc* OR streptococcus thermophilus OR bacillus subtilis OR enterococcus faec* OR bulgarian bacillus):ti,ab,kw

#29 #20 OR #21 OR #22 OR #23 OR #24 OR #25 OR #26 OR #27 OR #28

#30 MeSH descriptor: [Infant] explode all trees

#31 " MeSH descriptor: [Child] explode all trees

#32 MeSH descriptor: [Pediatrics] explode all trees

#33 (child* OR early life OR paediatric* OR pediatric* OR preschool* OR pre-school* OR infant*):ti,ab,kw

#34 #30 OR #31 OR #32 OR #33

#35 #19 AND #29 AND #34

Web of Science

#1 TS=(Respiratory Infection OR Upper Respiratory Infection OR Lower Respiratory Infection OR Respiratory Tract Infection OR Common Cold OR Sinusitis OR Pharyngitis OR Laryngitis OR Laryngotracheobronchitis OR Tonsillitis OR peritonsillar abscess OR Croup OR Epiglottitis OR supraglottitis OR Pneumonia OR Bronchopneumonia OR Bronchitis OR Pleurisy OR Pleuritis OR rhinitis OR rhinosinusitis)

#2 TS=(probiotic* OR lactobacill* OR bifido* OR bifidu* OR lactococc* OR saccharomyc* OR streptococcus thermophilus OR bacillus subtilis OR enterococcus faec* OR bulgarian bacillus)

#3 TS=(child* OR early life OR paediatric* OR pediatric* OR preschool* OR pre-school* OR infant*)

#4 TS=("randomized controlled trial" OR "Clinical trial" OR "Controlled Clinical trial" OR random* OR blind* OR singleblind* OR doubleblind* OR tripleblind*)

#5 #1 AND #2 AND #3 AND #4

CNKI

#1 SU = ('呼吸道感染' + '感冒' + '鼻窦炎' + '咽炎' + '喉炎' + '支气管炎' + '扁桃体炎' + '扁桃体周围脓肿' + '咳嗽' + '会厌炎' + '肺炎' + '支气管肺炎' + '胸膜炎' + '鼻炎') OR TKA = ('呼吸道感染' + '感冒' + '鼻窦炎' + '咽炎' + '喉炎' + '支气管炎' + '扁桃体炎' + '扁桃体周围脓肿' + '咳嗽' + '会厌炎' + '肺炎' + '支气管肺炎' + '胸膜炎' + '鼻炎')

#2 SU = ('益生菌' + '乳酸菌' + '双歧杆菌' + '乳球菌' + '糖酵母菌' + '嗜热链球菌' + '枯草杆菌' + '粪肠球菌' + '保加利亚杆菌') OR TKA = ('益生菌' + '乳酸菌' + '双歧杆菌' + '乳球菌' + '糖酵母菌' + '嗜热链球菌' + '枯草杆菌' + '粪肠球菌' + '保加利亚杆菌')

#3 TKA= (‘儿童’ + ‘小儿’ + ‘患儿’ + ‘婴儿’ + ‘学龄前’) OR SU= (‘儿童’ + ‘小儿’ + ‘患儿’ + ‘婴儿’ + ‘学龄前’)

#4 TKA= (‘随机’ + ‘安慰剂’ + ‘对照’ + ‘盲法’ + ‘单盲’ + ‘双盲’ + ‘三盲’) OR SU= (‘随机’ + ‘安慰剂’ + ‘对照’ + ‘盲法’ + ‘单盲’ + ‘双盲’ + ‘三盲’)

#5 #1 AND #2 AND #3 AND #4

WanFang

#1 全部:(呼吸道感染 or 感冒 or 鼻窦炎 or 咽炎 or 喉炎 or 支气管炎 or 扁桃体炎 or 扁桃体周围脓肿 or 咳嗽 or 会厌炎or 肺炎 or 支气管肺炎 or 胸膜炎 or 鼻炎)

#2 全部:(益生菌 or 乳酸菌 or 双歧杆菌 or 乳球菌 or 糖酵母菌 or 嗜热链球菌 or 枯草杆菌 or 粪肠球菌 or 保加利亚杆菌)

#3 全部: (儿童 or 小儿 or 患儿 or 婴儿 or 学龄前)

#4 全部: (随机 or 安慰剂 or 对照 or 盲法 or 单盲 or 双盲 or 三盲)

#5 #1 AND #2 AND #3 AND #4

CBM

#1 "呼吸道感染"[常用字段:智能] OR "感冒"[常用字段:智能] OR "鼻窦炎"[常用字段:智能] OR "咽炎"[常用字段:智能] OR "喉炎"[常用字段:智能] OR "支气管炎"[常用字段:智能] OR "扁桃体炎"[常用字段:智能] OR "扁桃体周围脓肿"[常用字段:智能] OR "咳嗽"[常用字段:智能] OR "会厌炎"[常用字段:智能] OR "肺炎"[常用字段:智能] OR "支气管肺炎"[常用字段:智能] OR "胸膜炎"[常用字段:智能] OR "鼻炎"[常用字段:智能]

#2 "益生菌"[常用字段:智能] OR "乳酸菌"[常用字段:智能] OR "双歧杆菌"[常用字段:智能] OR "乳球菌"[常用字段:智能] OR "糖酵母菌"[常用字段:智能] OR "嗜热链球菌"[常用字段:智能] OR "枯草杆菌"[常用字段:智能] OR "粪肠球菌"[常用字段:智能] OR "保加利亚杆菌"[常用字段:智能]

#3 ((#2) AND (#1)) AND ( 随机对照试验[文献类型]) AND ( "婴儿, 新生"[特征词] OR 婴儿[特征词] OR "儿童, 学龄前"[特征词] OR 儿童[特征词] OR 青少年[特征词]) AND ( 人类[特征词]

**Text S2 Definition of interested outcomes**

1. Duration of Core Symptoms: the number of calendar days from the initial recognition (or study enrolment confirmation) of a specific symptom to its documented resolution or disappearance.

1.1 Fever Duration: The time interval from the onset of febrile episode (temperature above a predefined threshold) to the point at which body temperature normalizes and remains stable without antipyretic support.

1.2 Cough Duration: The time interval from the onset of cough to its complete clinical resolution, defined as the absence of any cough episode for a consecutive period.

2. Duration of Illness: the number of calendar days from disease onset (or enrolment) to the attainment of clinical recovery, where "clinical recovery" is operationalized through one of four principal approaches across studies:

Symptom score‑based recovery: Complete resolution as reflected by a score of zero on a validated symptom instrument;

Prespecified clinical cure criterion: Complete disappearance of all disease‑related symptoms as determined by physician assessment or caregiver report, often assessed at fixed time points;

Total symptomatic days: The aggregate number of days during which any RTI‑related symptoms persist until complete resolution;

Proxy indicators: In certain studies, illness duration was represented by surrogate measures, length of hospital stay or duration of fever, rather than a composite multi‑symptom endpoint.

Core conceptual commonality: Despite differences in operationalization, the construct uniformly captures the complete temporal trajectory from illness onset to return to health, serving as a global measure of disease burden and recovery velocity.

3. Duration of Antibiotic Use: the cumulative number of calendar days during which systemic antibiotic therapy was administered to a child over a defined observation period.

Operational variations across studies:

Observation window: Per RTI episode, fixed timeframes of 7 or 21 days, or the entire follow‑up period including 12 months;

Unit of measurement: Reported either as mean days per episode or as total cumulative days per participant over the study period.

Core conceptual commonality: This endpoint consistently measures the total duration of antibiotic exposure, which serves as a proxy for both disease severity and the potential selective pressure favoring antimicrobial resistance.

4. Antibiotic Usage Rate: the proportion of participants within a given group who received at least one systemic antibiotic prescription during a specified observation period.

Operational variations across studies:

Observation timepoints: Assessed during the acute episode (7‑ or 21‑day windows), at the point of hospital discharge, during post‑discharge follow‑up, or across the entire study intervention period;

Reporting metric: Expresses the incidence proportion (percentage) of antibiotic users, rather than the frequency or duration of use.

Core conceptual commonality: This endpoint consistently provides a binary (yes/no) population‑level measure of antibiotic exposure, reflecting prescribing practices and serving as an important indicator for antimicrobial stewardship evaluation.

5. Adverse Event Incidence: the proportion of participants who experienced any untoward medical occurrence during the intervention period and follow‑up.

Standardized definitions:

Diarrhea: Passage of ≥3 loose or watery stools within a 24‑hour period;

Constipation: ≤2 defecations per week, pain/difficulty during defecation, fecal incontinence, or presence of large‑diameter stools obstructing the toilet;

Abdominal pain: As reported by the child to caregivers.

**Text S3 List of excluded studies**

Excluded because it was not a randomized controlled trial

[1] Mai TT, Thi Thu P, Thi Hang H, et al. Efficacy of probiotics on digestive disorders and acute respiratory infections: a controlled clinical trial in young Vietnamese children. Eur J Clin Nutr. 2021;75(3):513-520. doi:10.1038/s41430-020-00754-9
[2] Di Pierro F, Colombo M, Zanvit A, Rottoli AS. Positive clinical outcomes derived from using Streptococcus salivarius K12 to prevent streptococcal pharyngotonsillitis in children: a pilot investigation. Drug Healthc Patient Saf. 2016;8:77-81. doi:10.2147/DHPS.S117214
[3] Manti S, Parisi GF, Papale M, et al. Bacteriotherapy with Streptococcus salivarius 24SMB and Streptococcus oralis 89a nasal spray for treatment of upper respiratory tract infections in children: a pilot study on short-term efficacy. Ital J Pediatr. 2020;46(1):42. doi:10.1186/s13052-020-0798-4
[4] Santagati M, Scillato M, Muscaridola N, Metoldo V, La Mantia I, Stefani S. Colonization, safety, and tolerability study of the Streptococcus salivarius 24SMBc nasal spray for its application in upper respiratory tract infections. Eur J Clin Microbiol Infect Dis. 2015;34(10):2075-2080. doi:10.1007/s10096-015-2454-2
[5] Stojković A, Simović A, Bogdanović Z, Banković D, Poskurica M. Clinical trial/experimental study (consort compliant): Optimal time period to achieve the effects on synbiotic-controlled wheezing and respiratory infections in young children. Srp Arh Celok Lek. 2016;144(1-2):38-45. doi:10.2298/sarh1602038s

Excluded because data could not be extracted

[6] Tran DM, Tran TT, Phung TTB, et al. Nasal-spraying Bacillus spores as an effective symptomatic treatment for children with acute respiratory syncytial virus infection. Sci Rep. 2022;12(1):12402. doi:10.1038/s41598-022-16136-z
[7] Tran TT, Phung TTB, Tran DM, et al. Efficient symptomatic treatment and viral load reduction for children with influenza virus infection by nasal-spraying Bacillus spore probiotics. Sci Rep. 2023;13(1):14789. doi:10.1038/s41598-023-41763-5
[8] Campanella V, Syed J, Santacroce L, Saini R, Ballini A, Inchingolo F. Oral probiotics influence oral and respiratory tract infections in pediatric population: a randomized double-blinded placebo-controlled pilot study. Eur Rev Med Pharmacol Sci. 2018;22(22):8034-8041. doi:10.26355/eurrev_201811_16433
[9] Khieu TH, Le DP, Nguyen BT, et al. Alleviating symptoms of paediatric acute rhinosinusitis and acute otitis media with otorrhea using nasal-spraying Bacillus probiotics: a randomized controlled trial. Sci Rep. 2025;15(1):3410. Published 2025 Jan 27. doi:10.1038/s41598-025-87372-2

Excluded because the target population does not have Respiratory Tract Infections

[10] Hatakka K, Blomgren K, Pohjavuori S, et al. Treatment of acute otitis media with probiotics in otitis-prone children-a double-blind, placebo-controlled randomised study. Clin Nutr. 2007;26(3):314-321. doi:10.1016/j.clnu.2007.01.003
[11] Skovbjerg S, Roos K, Holm SE, et al. Spray bacteriotherapy decreases middle ear fluid in children with secretory otitis media. Arch Dis Child. 2009;94(2):92-98. doi:10.1136/adc.2008.137414
[12] Schrezenmeir J, Heller K, McCue M, et al. Benefits of oral supplementation with and without synbiotics in young children with acute bacterial infections. Clin Pediatr (Phila). 2004;43(3):239-249. doi:10.1177/000992280404300305
[13] Taipale TJ, Pienihäkkinen K, Isolauri E, Jokela JT, Söderling EM. Bifidobacterium animalis subsp. lactis BB-12 in reducing the risk of infections in early childhood. Pediatr Res. 2016;79(1-1):65-69. doi:10.1038/pr.2015.174
[14] Merenstein D, Murphy M, Fokar A, et al. Use of a fermented dairy probiotic drink containing Lactobacillus casei (DN-114 001) to decrease the rate of illness in kids: the DRINK study. A patient-oriented, double-blind, cluster-randomized, placebo-controlled, clinical trial. Eur J Clin Nutr. 2010;64(7):669-677. doi:10.1038/ejcn.2010.65
[15] Kanic Z, Micetic Turk D, Burja S, Kanic V, Dinevski D. Influence of a combination of probiotics on bacterial infections in very low birthweight newborns. Wien Klin Wochenschr. 2015;127 Suppl 5:S210-S215. doi:10.1007/s00508-015-0845-0
[16] Di Pierro F, Colombo M, Giuliani MG, et al. Effect of administration of Streptococcus salivarius K12 on the occurrence of streptococcal pharyngo-tonsillitis, scarlet fever and acute otitis media in 3 years old children. Eur Rev Med Pharmacol Sci. 2016;20(21):4601-4606.
[17] Hoy-Schulz YE, Jannat K, Roberts T, et al. Safety and acceptability of Lactobacillus reuteri DSM 17938 and Bifidobacterium longum subspecies infantis 35624 in Bangladeshi infants: a phase I randomized clinical trial. BMC Complement Altern Med. 2016;16:44. Published 2016 Feb 2. doi:10.1186/s12906-016-1016-1

Excluded because the primary intervention was medication

[18] Li B, Zheng J, Zhang X, Hong S. Probiotic Lactobacillus casei Shirota improves efficacy of amoxicillin-sulbactam against childhood fast breathing pneumonia in a randomized placebo-controlled double blind clinical study. J Clin Biochem Nutr. 2018;63(3):233-237. doi:10.3164/jcbn.17-117
[19] Santamaria F, Montella S, Stocchero M, et al. Effects of pidotimod and bifidobacteria mixture on clinical symptoms and urinary metabolomic profile of children with recurrent respiratory infections: a randomized placebo-controlled trial. Pulm Pharmacol Ther. 2019;58:101818. doi:10.1016/j.pupt.2019.101818

**Text S4-8 Result of certainty of evidence assessment**

Text S4 The certainty of evidence of duration of fever

| comparison | Direct Evidence | | | | | | | | | | | | | |
| --- | --- | --- | --- | --- | --- | --- | --- | --- | --- | --- | --- | --- | --- | --- |
|  | No of Study | No. of Participants | I² (%) | Egger’s value | MD (95%CI) | RoB | Inconsistency | Indirectness | Publication bias | Direct rating without imprecision | Imprecision | Direct rating with imprecision | Direct is more precise than indirect? | Need to rate indirect? |
| Control: Lactobacillus + Bifidobacterium | 2 | 172 | 97.60% | - | 4.50 (-27.89, 36.90) | Some concerns | Some concerns | No concerns | No concerns | Low | Some concerns | Very Low | Yes | No |
| Control: Bifidobacterium | 1 | 120 | - | - | 0.17 (-0.39, 0.73) | Some concerns | No concerns | No concerns | No concerns | Moderate | Some concerns | Low | Yes | No |
| Control: Lactobacillus + Bifidobacterium + Enterococcus + Bacillus | 1 | 60 | - | - | **1.54 (0.68, 2.40)** | No concerns | No concerns | No concerns | No concerns | High | Some concerns | Moderate | Yes | No |
| Lactobacillus + Bifidobacterium: Bifidobacterium | 0 | - | - | - | - | - | - | - | - | - | - | - | - | - |
| Lactobacillus + Bifidobacterium + Enterococcus + Bacillus: Bifidobacterium | 0 | - | - | - | - | - | - | - | - | - | - | - | - | - |
| Lactobacillus + Bifidobacterium: Lactobacillus + Bifidobacterium + Enterococcus + Bacillus | 0 | - | - | - | - | - | - | - | - | - | - | - | - | - |

| Indirect Evidence | | | | | | |  | Network Evidence | | | | | |
| --- | --- | --- | --- | --- | --- | --- | --- | --- | --- | --- | --- | --- | --- |
| MD (95%CI) | First order loop of the most contribution | Lowest of C1 and C2 | Intransitivity | Indirect rating without imprecision | Imprecision | Indirect rating with imprecision |  | MD (95%CI) | Incoherence, P-value | Higher rating of direct and indirect without imprecision | Incoherence | NMA Imprecision | Final network rating |
| - | - | - | - | - | - | - |  | 4.50 (-0.49, 9.50) | . | Low | No concerns | Some concerns | Very low |
| - | - | - | - | - | - | - |  | 0.17 (-6.84, 7.18) | . | Moderate | No concerns | Some concerns | Low |
| - | - | - | - | - | - | - |  | 1.54 (-5.50, 8.58) | . | High | No concerns | Some concerns | Moderate |
| -4.33 (-12.94, 4.27) | Control | Low | No concerns | Low | Some concerns | Very Low |  | -4.33 (-12.94, 4.27) | . | Low | No concerns | Some concerns | Very low |
| -1.37 (-11.30, 8.56) | Control | Moderate | No concerns | Moderate | Some concerns | Low |  | -1.37 (-11.30, 8.56) | . | Moderate | No concerns | Some concerns | Low |
| -2.96 (-11.59, 5.66) | Control | Low | No concerns | Low | Some concerns | Very Low |  | -2.96 (-11.59, 5.66) | . | Low | No concerns | Some concerns | Very low |

Text S5 The certainty of evidence of duration of cough

| comparison | Direct Evidence | | | | | | | | | | | | | |
| --- | --- | --- | --- | --- | --- | --- | --- | --- | --- | --- | --- | --- | --- | --- |
|  | No of Study | No. of Participants | I² (%) | Egger’s value | MD (95%CI) | RoB | Inconsistency | Indirectness | Publication bias | Direct rating without imprecision | Imprecision | Direct rating with imprecision | Direct is more precise than indirect? | Need to rate indirect? |
| Control: Lactobacillus + Bifidobacterium | 2 | 485 | 79.4 | - | 10.39 (-7.78, 28.56) | Some concerns | Some concerns | No concerns | No concerns | Low | Some concerns | Very Low | Yes | No |
| Control: Lactobacillus + Bifidobacterium + Enterococcus + Bacillus | 2 | 174 | 63 | - | 1.72 (-6.02, 9.46) | Some concerns | Some concerns | No concerns | No concerns | Low | Some concerns | Very Low | Yes | No |
| Control: Bifidobacterium | 1 | 120 | - | - | **0.85 (0.75, 0.95)** | No concerns | No concerns | No concerns | No concerns | High | No concerns | High | Yes | No |
| Lactobacillus + Bifidobacterium: Bifidobacterium | 0 | - | - | - | - | - | - | - | - | - | - | - | - | - |
| Lactobacillus + Bifidobacterium + Enterococcus + Bacillus: Bifidobacterium | 0 | - | - | - | - | - | - | - | - | - | - | - | - | - |
| Lactobacillus + Bifidobacterium: Lactobacillus + Bifidobacterium + Enterococcus + Bacillus | 0 | - | - | - | - | - | - | - | - | - | - | - | - | - |

| Indirect Evidence | | | | | | |  | Network Evidence | | | | | |
| --- | --- | --- | --- | --- | --- | --- | --- | --- | --- | --- | --- | --- | --- |
| MD (95%CI) | First order loop of the most contribution | Lowest of C1 and C2 | Intransitivity | Indirect rating without imprecision | Imprecision | Indirect rating with imprecision |  | MD (95%CI) | Incoherence, P-value | Higher rating of direct and indirect without imprecision | Incoherence | NMA Imprecision | Final network rating |
| - | - | - | - | - | - | - |  | **10.63 (8.75, 12.52)** | . | Low | No concerns | No concerns | Low |
| - | - | - | - | - | - | - |  | **1.80 (0.11, 3.48)** | . | Low | No concerns | No concerns | Low |
| - | - | - | - | - | - | - |  | 0.85 (-1.32, 3.02) | . | High | No concerns | Some concerns | Moderate |
| **-9.78 (-12.66, -6.91)** | Control | Low | No concerns | Low | No concerns | Low |  | **-9.78 (-12.66, -6.91)** | . | Low | No concerns | No concerns | Low |
| -0.95 (-3.69, 1.80) | Control | Low | No concerns | Low | Some concerns | Very Low |  | -0.95 (-3.69, 1.80) | . | Low | No concerns | Some concerns | Very low |
| **-8.84 (-11.37, -6.31)** | Control | Low | No concerns | Low | No concerns | Low |  | **-8.84 (-11.37, -6.31)** | . | Low | No concerns | No concerns | Low |

Text S6 The certainty of evidence of duration of illness

| comparison | Direct Evidence | | | | | | | | | | | | | |
| --- | --- | --- | --- | --- | --- | --- | --- | --- | --- | --- | --- | --- | --- | --- |
|  | No of Study | No. of Participants | I² (%) | Egger’s value | MD (95%CI) | RoB | Inconsistency | Indirectness | Publication bias | Direct rating without imprecision | Imprecision | Direct rating with imprecision | Direct is more precise than indirect? | Need to rate indirect? |
| Lactobacillus + Bifidobacterium: Control | 3 | 622 | 99.9 | - | -11.07 (-34.79, 12.65) | Some concerns | Some concerns | No concerns | No concerns | Low | Some concerns | Very Low | Yes | No |
| Lactobacillus: Control | 2 | 167 | 0 | - | **-1.33 (-1.65, -1.02)** | Some concerns | No concerns | No concerns | No concerns | Moderate | No concerns | Moderate | Yes | No |
| Lactobacillus: Lactobacillus + Bifidobacterium | 0 | - | - | - | - | - | - | - | - | - | - | - | - | - |

| Indirect Evidence | | | | | | |  | Network Evidence | | | | | |
| --- | --- | --- | --- | --- | --- | --- | --- | --- | --- | --- | --- | --- | --- |
| MD (95%CI) | First order loop of the most contribution | Lowest of C1 and C2 | Intransitivity | Indirect rating without imprecision | Imprecision | Indirect rating with imprecision |  | MD (95%CI) | Incoherence, P-value | Higher rating of direct and indirect without imprecision | Incoherence | NMA Imprecision | Final network rating |
| - | - | - | - | - | - | - |  | -11.07 (-23.82, 1.68) | - | Low | No concerns | Some concerns | Very low |
| - | - | - | - | - | - | - |  | -1.33 (-16.92, 14.25) | - | Moderate | No concerns | Some concerns | Low |
| 9.73 (-10.40, 29.87) | Control | Low | No concerns | Low | Some concerns | Very Low |  | 9.73 (-10.40, 29.87) | - | Low | No concerns | Some concerns | Very low |

Text S7 The certainty of evidence of duration of antibiotic use

| comparison | Direct Evidence | | | | | | | | | | | | | |
| --- | --- | --- | --- | --- | --- | --- | --- | --- | --- | --- | --- | --- | --- | --- |
|  | No of Study | No. of Participants | I² (%) | Egger’s value | MD (95%CI) | RoB | Inconsistency | Indirectness | Publication bias | Direct rating without imprecision | Imprecision | Direct rating with imprecision | Direct is more precise than indirect? | Need to rate indirect? |
| Lactobacillus: Control | 2 | 167 | 83.2 | - | -1.07 (-9.20, 7.06) | Some concerns | Some concerns | No concerns | No concerns | Low | Some concerns | Very Low | Yes | No |
| Lactobacillus + Bifidobacterium: Control | 1 | 85 | - | - | **-10.50 (-13.22, -7.78)** | Some concerns | No concerns | No concerns | No concerns | Moderate | Some concerns | Low | Yes | No |
| Lactobacillus: Lactobacillus + Bifidobacterium | 0 | - | - | - | - | - | - | - | - | - | - | - | - | - |

| Indirect Evidence | | | | | | |  | Network Evidence | | | | | |
| --- | --- | --- | --- | --- | --- | --- | --- | --- | --- | --- | --- | --- | --- |
| MD (95%CI) | First order loop of the most contribution | Lowest of C1 and C2 | Intransitivity | Indirect rating without imprecision | Imprecision | Indirect rating with imprecision |  | MD (95%CI) | Incoherence, P-value | Higher rating of direct and indirect without imprecision | Incoherence | NMA Imprecision | Final network rating |
| - | - | - | - | - | - | - |  | -1.07 (-2.32, 0.19) | - | Low | No concerns | Some concerns | Very low |
| - | - | - | - | - | - | - |  | **-10.50 (-13.67, -7.33)** | - | Moderate | No concerns | No concerns | Moderate |
| 9.43 (6.04, 12.84) | Control | Low | No concerns | Low | No concerns | Low |  | **9.43 (6.03, 12.84)** | - | Low | No concerns | No concerns | Low |

Text S8 The certainty of evidence of antibiotic usage rate

| comparison | Direct Evidence | | | | | | | | | | | | | |
| --- | --- | --- | --- | --- | --- | --- | --- | --- | --- | --- | --- | --- | --- | --- |
|  | No of Study | No. of Participants | I² (%) | Egger’s value | OR (95%CI) | RoB | Inconsistency | Indirectness | Publication bias | Direct rating without imprecision | Imprecision | Direct rating with imprecision | Direct is more precise than indirect? | Need to rate indirect? |
| Control: Lactobacillus | 2 | 167 | 67.7 | - | 2.62 (0.00, 37073.97) | Some concerns | Some concerns | No concerns | No concerns | Low | Some concerns | Very Low | Yes | No |
| Control: Bifidobacterium | 1 | 120 | 0 | - | 1.11 (0.46, 2.67) | Some concerns | No concerns | No concerns | No concerns | Moderate | Some concerns | Low | Yes | No |
| Lactobacillus: Bifidobacterium | 0 | - | - | - | - | - | - | - | - | - | - | - | - | - |

| Indirect Evidence | | | | | | |  | Network Evidence | | | | | |
| --- | --- | --- | --- | --- | --- | --- | --- | --- | --- | --- | --- | --- | --- |
| OR (95%CI) | First order loop of the most contribution | Lowest of C1 and C2 | Intransitivity | Indirect rating without imprecision | Imprecision | Indirect rating with imprecision |  | OR (95%CI) | Incoherence, P-value | Higher rating of direct and indirect without imprecision | Incoherence | NMA Imprecision | Final network rating |
| - | - | - | - | - | - | - |  | 2.62 (0.60, 11.46) | - | Low | No concerns | Some concerns | Very low |
| - | - | - | - | - | - | - |  | 1.11 (0.16, 7.69) | - | Moderate | No concerns | Some concerns | Low |
| 2.37 (0.21, 26.50) | Control | Low | No concerns | Low | Some concerns | Very Low |  | 2.37 (0.21, 26.50) | - | Low | No concerns | Some concerns | Very low |

**Table S1 Results of risk of bias assessment**

| **ID** | **Sequence generation** | **Allocation concealment** | **Blinding of participants and personnel** | **Blinding of outcome assessors** | **Incomplete outcome data** | **Selective outcome reporting** | **Other sources of bias** |
| --- | --- | --- | --- | --- | --- | --- | --- |
| Gerasimov, 2016^[23]^ | Low | Low | Low | Low | High | Low | Low |
| Huo, 2018^[24]^ | Unclear | Unclear | Low | Low | Low | Low | Low |
| Li, 2019^[25]^ | Low | Unclear | Unclear | Unclear | Low | Low | Low |
| Mageswary, 2022^[26]^ | Low | Low | Low | Low | Low | Low | Low |
| Zhang, 2022^[27]^ | Low | Unclear | Unclear | Unclear | Low | Low | Unclear |
| Pulmamidi, 2023^[28]^ | Unclear | Unclear | Low | Low | Low | Low | Unclear |
| He, 2024^[29]^ | Low | Unclear | Unclear | Unclear | Low | Low | Unclear |
| Bettocchi, 2025^[30]^ | Low | Low | Low | Low | High | Low | Low |

**Table S2-6 The results of sensitivity analysis by using different models**

Table S2 The result of sensitivity analysis of duration of fever

|  | DL model without HKSJ | | | |
| --- | --- | --- | --- | --- |
| Fixed effect model | Bifidobacterium | -0.17 (-7.18, 6.84) | 4.33 (-4.27, 12.94) | 1.37 (-8.56, 11.30) |
|  | -0.17 (-0.73, 0.39) | Control | 4.50 (-0.49, 9.50) | 1.54 (-5.50, 8.58) |
|  | **2.51 (1.74, 3.28)** | **2.68 (2.15, 3.20)** | Lactobacillus + Bifidobacterium | -2.96 (-11.59, 5.66) |
|  | **1.37 (0.34, 2.40)** | **1.54 (0.68, 2.40)** | **-1.14 (-2.15, -0.13)** | Lactobacillus + Bifidobacterium + Enterococcus + Bacillus |

Table S3 The result of sensitivity analysis of duration of cough

|  | DL model without HKSJ | | | |
| --- | --- | --- | --- | --- |
| Fixed effect model | Bifidobacterium | -0.85 (-3.02, 1.32) | **9.78 (6.91, 12.66)** | 0.95 (-1.80, 3.69) |
|  | **-0.85 (-0.95, -0.75)** | Control | **10.63 (8.75, 12.52)** | **1.80 (0.11, 3.48)** |
|  | **10.47 (9.69, 11.24)** | **11.32 (10.55, 12.08)** | Lactobacillus + Bifidobacterium | **-8.84 (-11.37, -6.31)** |
|  | **0.59 (0.04, 1.14)** | **1.44 (0.90, 1.98)** | **-9.87 (-10.81, -8.93)** | Lactobacillus + Bifidobacterium + Enterococcus + Bacillus |

Table S4 The result of sensitivity analysis of duration of illness

|  | DL model without HKSJ | | |
| --- | --- | --- | --- |
| Fixed effect model | Control | 1.33 (-14.25, 16.92) | 11.07 (-1.68, 23.82) |
|  | **1.33 (0.70, 1.97)** | Lactobacillus | 9.73 (-10.40, 29.87) |
|  | **6.09 (5.77, 6.41)** | **4.76 (4.05, 5.47)** | Lactobacillus + Bifidobacterium |

Table S5 The result of sensitivity analysis of duration of antibiotic use

|  | DL model without HKSJ | | |
| --- | --- | --- | --- |
| Fixed effect model | Control | 1.07 (-0.19, 2.32) | **10.50 (7.33, 13.67)** |
|  | **1.11 (0.59, 1.62)** | Lactobacillus | **9.43 (6.03, 12.84)** |
|  | **10.50 (7.78, 13.22)** | **9.39 (6.62, 12.16)** | Lactobacillus + Bifidobacterium |

Table S6 The result of sensitivity analysis of antibiotic usage rate

|  | DL model without HKSJ | | |
| --- | --- | --- | --- |
| Fixed effect model | Bifidobacterium | 0.90 (0.13, 6.11) | 2.37 (0.21, 26.50) |
|  | 0.90 (0.37, 2.18) | Control | 2.62 (0.60, 11.46) |
|  | 1.84 (0.57, 5.92) | 2.03 (0.94, 4.39) | Lactobacillus |

**Table S7-9 The results of sensitivity analysis by excluding studies with attrition rates exceeding 20%**

Table S7 The result of sensitivity analysis of duration of fever

| Bifidobacterium |  |  |  |
| --- | --- | --- | --- |
| -0.17 (-0.73, 0.39) | Control |  |  |
| **6.93 (5.38, 8.48)** | **7.10 (5.66, 8.54)** | Lactobacillus + Bifidobacterium |  |
| **1.37 (0.34, 2.40)** | **1.54 (0.68, 2.40)** | **-5.56 (-7.24, -3.88)** | Lactobacillus + Bifidobacterium + Enterococcus + Bacillus |

Table S8 The result of sensitivity analysis of duration of cough

| Bifidobacterium |  |  |  |
| --- | --- | --- | --- |
| -0.85 (-2.24, 0.54) | Control |  |  |
| **7.85 (4.71, 10.99)** | **8.70 (5.89, 11.51)** | Lactobacillus + Bifidobacterium |  |
| 0.87 (-0.96, 2.70) | **1.72 (0.52, 2.91)** | **-6.98 (-10.04, -3.93)** | Lactobacillus + Bifidobacterium + Enterococcus + Bacillus |

Table S9 The result of sensitivity analysis of duration of illness

| Control |  |  |
| --- | --- | --- |
| **1.33 (0.70, 1.97)** | Lactobacillus |  |
| **10.20 (7.24, 13.16)** | **8.87 (5.84, 11.90)** | Lactobacillus + Bifidobacterium |

**Figure S1-5 Forest plot of pairwise meta-analysis**

Figure S1 Forest plot of duration of fever (days)

Figure S2 Forest plot of duration of cough (days)

Figure S3 Forest plot of duration of illness (days)

Figure S4 Forest plot of duration of antibiotic use (days)

Figure S5 Forest plot of antibiotic usage rate

**Figure S6-10 Network plot of network meta-analysis**

Figure S6 Network plot of duration of fever

Figure S7 Network of duration of cough

Figure S8 Network of duration of illness

Figure S9 Network of duration of antibiotic use

Figure S10 Network of antibiotic usage rate
